# Phytochemical Profiling and In Vitro Antibacterial and Antileishmanial Activities of *Flourensia cernua*, *Artemisia ludoviciana*, and *Phoradendron californicum* Extracts Obtained by Ultrasound-Assisted Extraction

**DOI:** 10.3390/cimb48101017

**Published:** 2026-10-01

**Authors:** Antonio Julián-Flores, Pedro Aguilar-Zárate, Cristóbal N. Aguilar, Mariela R. Michel, Teresinha Gonçalves da Silva, Juan A. Ascacio-Valdés, Mónica L. Chávez-González

**Affiliations:** 1 Bioprocesses & Bioproducts Group, Food Research Department, School of Chemistry, Universidad Autónoma de Coahuila, Saltillo 25280, Coahuila, Mexico; antoniojulian@uadec.edu.mx (A.J.-F.); cristobal.aguilar@uadec.edu.mx (C.N.A.); alberto_ascaciovaldes@uadec.edu.mx (J.A.A.-V.); 2 Laboratorio Nacional CONAHCYT de Apoyo a la Evaluación de Productos Bióticos (LaNAEPBi), Unidad de Servicio, Tecnológico Nacional de México/I.T. de Ciudad Valles, Ciudad Valles 79010, San Luis Potosí, Mexico; mariela.michel@tecvalles.mx; 3 Department of Antibiotics, Federal University of Pernambuco (UFPE), Recife 54740-520, Pernambuco, Brazil; teresinha.goncalves@ufpe.br

**Keywords:** medicinal plants, ultrasound-assisted extraction, bioactive compounds, antioxidant activity, antimicrobial activity

## Abstract

Medicinal plants are a valuable source of bioactive compounds with a broad range of therapeutic properties. In Mexico, the bioactive potential of *Flourensia cernua*, *Artemisia ludoviciana*, and *Phoradendron californicum* continues to be investigated; however, conventional extraction approaches often result in low yields of bioactive compounds. The aim of this study was to optimize the extraction process using the Taguchi methodology coupled with ultrasound-assisted extraction. An L9 (3^3^) orthogonal array was employed to evaluate extraction time, ethanol concentration, and solid-to-liquid ratio, using total phenolic content as the response variable. Under the optimal conditions predicted by the software, condensed tannins, total flavonoids, and antioxidant, antiparasitic, and antibacterial activities were subsequently determined. The compounds present in the extracts were tentatively identified by HPLC–MS. *P. californicum* exhibited the highest levels of total phenolic content and total flavonoids, with 106.07 ± 4.16 GAE mg∙g^−1^ and 9.04 ± 0.31 CE mg∙g^−1^, respectively. *F. cernua* showed the highest content of condensed tannins, reaching 6.71 ± 0.10 CE mg∙g^−1^. ABTS radical-scavenging activity was highest for *A. ludoviciana* (IC_50_ = 19.37 ± 4.23 μg∙mL^−1^), whereas *F. cernua* exhibited the highest DPPH radical-scavenging activity (IC_50_ = 755.92 ± 70.60 μg∙mL^−1^). Furthermore, *A. ludoviciana* showed the highest antiparasitic activity (IC_50_ = 0.51 ± 0.20 mg∙mL^−1^) and antibacterial activity against the pathogenic bacteria *Escherichia coli* (89.59%) and *Staphylococcus aureus* (91.55%). These findings support the potential of UAE-optimized extracts as sources of bioactive ingredients for industrial applications.

## 1. Introduction

In recent years, interest in consuming natural plant-based products has grown, driven mainly by bioactive compounds with potential health benefits. These compounds represent an important source for the development of food products, nutraceuticals, cosmetics, and pharmaceuticals [1]. Among the main plant-derived bioactive compounds, polyphenols are particularly relevant due to their diverse biological properties, including antioxidant, anti-inflammatory, anticancer, and antimicrobial activities [2]. From a plant physiology perspective, polyphenols are secondary metabolites that play important roles in plant–environment interactions. Their biosynthesis and accumulation are closely associated with the environmental conditions under which plants develop, as they contribute to adaptive and defense mechanisms against various stressors, including insects and microorganisms such as fungi [3].

In the semiarid regions of Mexico, a remarkable diversity of plant species has adapted to extreme environmental conditions, characterized by low precipitation and high temperatures. These conditions may promote the biosynthesis and accumulation of bioactive compounds with potential therapeutic properties [4]. Species of particular interest include *Flourensia cernua*, *Artemisia ludoviciana*, and *Phoradendron californicum*, which have traditionally been used to treat various health conditions. Conventional extraction methods, such as maceration, percolation, and infusion, have been employed to recover bioactive compounds from these plants; however, these approaches may result in low extraction yields and limit the recovery of target compounds [5,6]. In response to these limitations, novel extraction technologies have been implemented, including cold atmospheric plasma extraction, pressurized liquid extraction, supercritical fluid extraction, microwave-assisted extraction (MAE), and ultrasound-assisted extraction (UAE). Among these approaches, UAE is one of the most widely used techniques for the recovery of bioactive compounds due to its effectiveness and potential to enhance extraction yields [7]. UAE extraction is considered a sustainable technology that uses high-intensity ultrasonic waves to induce acoustic cavitation, thereby facilitating the release and extraction of bioactive compounds. In addition, this technique can reduce processing time and improve extraction efficiency and reproducibility, while enhancing the quality and yield of the resulting extracts [8,9]. The efficiency of the extraction process is influenced by several factors, including the type and concentration of the solvent, solid-to-liquid ratio, extraction temperature, ultrasonic frequency, and particle size of the plant material [10]. Therefore, systematic optimization of the extraction process is essential for the efficient recovery of bioactive compounds while maintaining the quality of plant-based products. However, process optimization can require considerable time, energy, and resources. To address this complexity, various mathematical and statistical models have been developed and applied to simultaneously evaluate multiple factors influencing the extraction process [11]. Among these tools, the Taguchi methodology is a robust optimization technique that enables the simultaneous evaluation of multiple factors using a limited number of experiments. This approach employs orthogonal arrays to identify the optimal process conditions while reducing the experimental workload [12]. Currently, reports on the optimization of bioactive compound recovery by UAE from plant species native to the Mexican semidesert are limited. Nevertheless, the feasibility of this technique for extracting bioactive compounds from *F. cernua* under different extraction conditions has been demonstrated, highlighting its potential to promote the release of phytochemicals present in this species. Therefore, this study aimed to optimize the extraction of phytochemicals from *F. cernua*, *A. ludoviciana*, and *P. californicum* using UAE coupled with a Taguchi L9 design and to evaluate the antioxidant and antimicrobial effects of the resulting extracts.

## 2. Materials and Methods

### 2.1. Plant Material

*Flourensia cernua* and *Phoradendron californicum* were harvested in Parras, Coahuila, Mexico (25°33′51.5″ N, 102°33′54.5″ W) during November and December 2023. *Artemisia ludoviciana* was obtained from a local herbal supplier (Centro Botánico Azteca, Saltillo, Coahuila, Mexico) in December 2023. Botanical identification of all plant materials was performed by the Research Center and Ethnobiological Garden (CIJE). Leaves from *F. cernua* and *P. californicum* were selected for subsequent analyses, whereas the entire aerial portion of *A. ludoviciana* was processed. Prior to extraction, the plant material was dried at 60 °C for 24 h a NAPCO 1000 incubator (National Appliance Co., Portland, OR, USA) and then ground with a mortar.

### 2.2. Taguchi L9 Orthogonal Array

The Taguchi methodology was used to optimize the extraction process for total phenolic content (TPC). Three factors—extraction time, ethanol concentration, and solid/liquid ratio—were examined at three levels each (Table 1). An L9 (3^3^) orthogonal array was generated, producing nine experimental runs corresponding to the three factors and their three levels (Table 2).

In this study, the “higher-the-better” quality characteristic was selected from among the three categories (“smaller-the-better,” “higher-the-better,” and “nominal-the-best”) of the Taguchi methodology. It is expressed as L(y) = k × (1/y^2^) and is represented as follows:

(1) $$E\left[L(y)\right]=-10*\log_{10}\left[\left(1/n)*\sum (1/y_{i}^{2}\right)\right]$$

where the factor −10 ensures that this ratio measures the inverse of “poor quality,” and *n* represents the number of samples. Following the analysis of variance (ANOVA), the percentage contribution of each source of variation was calculated as follows:

(2) $$P=\frac{{\mathrm{SS}}_{i}}{{\mathrm{SS}}_{T}}\times 100\%=\frac{{\mathrm{SS}}_{i}-{\mathrm{MS}}_{i}*{\mathrm{df}}_{i}}{{\mathrm{SS}}_{T}}*100\%$$

where *P* is the contribution percentage, *SS_i_* is the individual sum of squares, *SS_T_* is the total sum of squares, *MS_i_* is the initial mean square, and *df_i_* represents the individual degrees of freedom. The experimental data were analyzed using Statistica software, version 7.0 (StatSoft, Tulsa, OK, USA).

### 2.3. Ultrasound-Assisted Extraction and Validation

Plant extraction was performed by transferring 0.5 g of powdered plant material into a test tube, followed by the addition of the extraction solvent according to the solvent composition and solid/liquid ratio specified in the experimental design (Table 1 and Table 2). The mixtures were sonicated in an ultrasonic bath (CPX3800H, BRANSON, Brookfield, CT, USA) at 40 kHz for the extraction time defined by the orthogonal array. Following extraction, the samples were filtered through Whatman No. 1 filter paper. All extraction experiments were performed in triplicate.

The optimal extraction conditions for the compounds in the extracts were experimentally validated by quantifying the total phenolic content. The experimental and predicted values were compared to assess the validity of the model. Subsequently, under these extraction conditions, the condensed tannin content, total flavonoid content, antioxidant activity, and antimicrobial and antiparasitic activities were determined.

### 2.4. Total Phenolic Content

Total phenolic content was quantified according to the Folin–Ciocalteu method described by Torres-León et al. [13], with minor modifications. Gallic acid was used as a standard solution (0–200 ppm). Samples were diluted (1:10, *v*/*v*), and 20 µL of the diluted sample was mixed with 20 µL of Folin–Ciocalteu reagent and incubated at room temperature for 5 min. Subsequently, 20 µL of 0.01 M sodium carbonate solution was added and incubated for an additional 5 min. Then, 125 µL of distilled water was added, and the absorbance was measured at 734 nm using a microplate reader.

### 2.5. Condensed Tannins Content

Condensed tannins were quantified according to the HCl–butanol method. A catechin standard solution (0–1000 ppm) was used for calibration. Samples were diluted (1:10, *v*/*v*), and 500 µL of the diluted extract was mixed with 3 mL of HCl–butanol solution (1:9, *v*/*v*) and 100 µL of ferric reagent. The mixtures were incubated in a water bath at 100 °C for 1 h. Absorbance was measured at 460 nm using a microplate reader [13].

### 2.6. Total Flavonoid Content

Total flavonoid content was determined according to the method described by De la Rosa et al. [14], with minor modifications. A catechin standard solution (100–1000 ppm) was used for calibration. Briefly, 310 µL of the extract was mixed with 93 µL of 5% NaNO_3_ and 93 µL of distilled water and incubated for 5 min. Subsequently, 93 µL of 10% AlCl_3_ was added and incubated for 3 min. Then, 125 µL of 0.5 M NaOH was added, and the mixture was incubated at room temperature for 30 min in the dark. Absorbance was measured at 510 nm using a spectrophotometer (BIOBASE-EL10A, Biobase Biodustry (Shandong Co., Ltd., Jinan, China). All assays were performed in triplicate.

### 2.7. Antioxidant Activity

#### 2.7.1. ABTS

ABTS•+ radical scavenging activity was determined according to the method described by Aranda-Ledezma et al. [15], with minor modifications. The ABTS•+ radical cation was prepared by reacting 7 mmol·L^−1^ ABTS with 2.45 mmol·L^−1^ potassium persulfate and incubating the mixture in the dark at room temperature for 12 h. The working solution was adjusted with ethanol to an absorbance of 0.700 ± 0.002 at 734 nm. Subsequently, 5 µL of the sample was mixed with 95 µL of the ABTS•+ solution, and the absorbance was measured at 734 nm after 1 min of reaction. The antioxidant activity of plant extracts was expressed as IC_50_. The inhibition values were plotted against extract concentration (concentration range of 50–0.097 mg/mL), and the IC_50_ was calculated from the corresponding regression equation as the concentration of extract required to inhibit 50% of the ABTS radicals. Trolox was used as a reference antioxidant.

#### 2.7.2. DPPH

DPPH radical scavenging activity was determined according to the method described by Siller-Sánchez et al. [16], with minor modifications. A 60 µM DPPH solution was prepared in methanol. Then, 7 µL of the sample was mixed with 193 µL of the DPPH solution in a 96-well microplate and incubated at room temperature for 30 min. Absorbance was measured at 517 nm using a microplate reader (BIOBASE-EL10A, Biobase Biodustry (Shandong) Co., Ltd., Jinan, China). The antioxidant activity of plant extracts was expressed as IC_50_. The inhibition values were plotted against extract concentration (concentration range of 50–0.097 mg/mL), and the IC_50_ was calculated from the corresponding regression equation as the concentration of extract required to inhibit 50% of the DPPH radicals.

### 2.8. Antimicrobial Activity

#### 2.8.1. Antileishmanial Activity

The antiparasitic activity of the extracts against *Leishmania braziliensis* LTB (MHOM/BR/75/M2903) promastigotes (10^6^ cells/mL) was evaluated according to Pereira et al. [17], with minor modifications. Briefly, 50 µL of Schneider’s medium, 50 µL of the sample (initial concentration of 50 mg/mL, serially diluted), and 50 µL of parasite suspension were added to each well of a 96-well microplate. The plates were incubated at 26 °C for 24 h, and cell growth was determined by counting in a Neubauer chamber. The percentage of growth inhibition was calculated for each extract concentration relative to the untreated control (Equation (3)). The resulting concentration–response data were used to construct a regression curve, and linear regression was applied to the concentration range showing an approximately linear relationship between extract concentration and percentage of inhibition. The IC_50_ was estimated from the resulting regression equation as the extract concentration corresponding to 50% inhibition of promastigote growth. Assays were performed in quintuplicate in two independent experiments.

(3) $$\mathrm{Relative}\;\mathrm{growth}\;(\%)=\frac{\mathrm{Paratises}\;\mathrm{in}\;\mathrm{sample}}{\mathrm{Parasites}\;\mathrm{in}\;\mathrm{untreated}\;\mathrm{control}}\times 100\%$$

#### 2.8.2. Antibacterial Activity

The antibacterial activity of the extracts against *Escherichia coli* (ATCC 25922) and *Staphylococcus aureus* (ATCC 25923) was evaluated using the broth microdilution method described by Haq et al. [18]. Briefly, 100 µL of Mueller–Hinton broth was added to each well of the 96-well microdilution tray. Then, 100 µL of the extract was added to each well, except for those that served as sterile and positive controls. Subsequently, 10 µL of bacterial suspension (2 × 10^5^ CFU/mL) was added to each well except for the sterile control well, resulting in a final inoculum of approximately 9.5 × 10^3^ CFU/mL and a final well volume of 210 µL. The initial concentration of the extract was 50 mg/mL, which was serially diluted (1:2) to a concentration of 0.097 mg/mL. Tetracycline (128–0.5 µg/mL) was used as the positive control according to the Clinical and Laboratory Standards Institute (CLSI M100, 2021) guidelines. The plates were incubated at 37 °C for 18–24 h, and the minimum inhibitory concentration (MIC) was determined as the lowest concentration of the extract that inhibited visible bacterial growth.

##### Spectrophotometric Broth Microdilution Method

Bacterial growth inhibition was additionally evaluated using the spectrophotometric broth microdilution method. Optical density (OD) was measured at 620 nm using a microplate reader (BIOBASE-EL10A, Biobase Biodustry (Shandong) Co., Ltd., Jinan, China) before incubation (t_0_) and after 24 h of incubation (t_24_). Growth inhibition was calculated from the OD values, where negative values were set to zero, and values exceeding 100 were assigned as 100. Values outside the 0–100 range were considered non-interpretable: negative values can arise when the treated sample’s apparent growth exceeds that of the control, while values above 100% may result from differences in optical density readings relative to the control. Growth inhibition was calculated according to the following equation:

(4) $$\mathrm{Grown}\;\mathrm{Inhibition}\;\left(\%\right)=100-\frac{\left({\mathrm{OD}\;\mathrm{test}\;t}_{24}-t_{0}\right)}{\left({\mathrm{OD}\;\mathrm{control}\;t}_{24}-t_{0}\right)}\times 100\;$$

where OD test refers to the extract sample at 0 and 24 h, and OD control refers to the medium containing bacteria at 0 and 24 h.

### 2.9. Identification of Phytochemical Compounds via HPLC-MS

Plant extracts were characterized by high-performance liquid chromatography and mass spectroscopy according to the methodology described by Ascacio-Valdés et al. [19]. The system consisted of an autosampler (Varian ProStar 410, Palo Alto, CA, USA), a ternary pump (Varian ProStar 230I, Palo Alto, CA, USA), and a PDA detector (Varian ProStar 330, Atlanta, CA, USA). A liquid chromatography ion trap mass spectrometer (Varian 500-MS IT mass spectrometer, Palo Alto, CA, USA) was also utilized with an electrospray ion source. The samples (5 µL) were injected onto a Denali C18 column (150 × 2.1 mm, 3 µm, Grace, Palo Alto, CA, USA). The oven temperature was maintained at 30 °C. The eluents were formic acid (0.2%, *v*/*v*; solvent A) and acetonitrile (solvent B). The following gradient was applied: initial, 3% B; 0–5 min, 9% B linear; 5–15 min, 16% B linear; and 15–45 min, 50% B linear. The column was then washed and reconditioned. The flow rate was maintained at 0.2 mL min^−1^, and elution was monitored at 254, 280, 320, and 550 nm. The entire effluent (0.2 mL min^−1^) was injected into the mass spectrometer source without splitting. All MS experiments were carried out in negative mode [M−H]^−^. Nitrogen was used as the nebulizing gas, and helium was used as the damping gas. The ion source parameters were as follows: spray voltage 5.0 kV; capillary voltage 90.0 V; and capillary temperature 350 °C. The data were collected and processed via MS Workstation software (V 6.9). The samples were first analyzed in full-scan mode in the *m*/*z* range of 50–2000. Putative compounds were assigned by comparing the observed *m*/*z* values and retention times with available literature and database records. Because authentic analytical standards were not used for confirmation, the detected compounds were considered tentatively identified.

### 2.10. Statistical Analysis

All experiments were performed using independent biological replicates. Antileishmanial activity was evaluated in quintuplicate in two independent experiments, whereas antibacterial activity was evaluated in triplicate in two independent experiments. Data were expressed as mean ± standard deviation (SD). For both biological assays, differences among plant extracts were evaluated by one-way analysis of variance (ANOVA), followed by Tukey’s honestly significant difference (HSD) test for multiple mean comparisons at a significance level of *p* < 0.05. All analyses were conducted using Statistica software, version 7.0 (StatSoft, Tulsa, OK, USA).

## 3. Results

### 3.1. Extraction Process Optimization

Table 2 presents the results of the nine treatments in the Taguchi orthogonal array. These results were used to perform the analysis of variance (ANOVA) and determine the contribution and effect of each factor and its corresponding levels on TPC extraction. Based on these analyses, the optimal extraction conditions for maximizing TPC yield were subsequently determined.

The validation of the extraction conditions predicted by the software is presented in Table 3. For *F. cernua*, a TPC of 53.66 mg GAE·g^−1^ was predicted under extraction conditions of 10 min, 35% ethanol, and a solid/liquid ratio of 1:5 (*w*/*v*). For *A. ludoviciana*, the predicted conditions consisted of an extraction time of 20 min, an ethanol concentration of 35%, and a solid/liquid ratio of 1:20 (*w*/*v*), with a predicted TPC of 15.85 mg GAE·g^−1^. For *P. californicum*, the predicted TPC was 87.33 mg GAE·g^−1^ with 20 min of sonication, using distilled water (0% ethanol) and a solid/liquid ratio of 1:20 (*w*/*v*). These values corresponded to differences of 6.97%, 12.37%, and 21.47% relative to the predicted values, respectively.

The total flavonoid and condensed tannin contents obtained under the optimal extraction conditions are presented in Table 4. Among the evaluated species, *P. californicum* exhibited the highest total flavonoid content (9.04 ± 0.31 mg CE/g), whereas *F. cernua* showed the highest condensed tannin content (6.71 ± 0.10 mg CE/g). According to Tukey’s test, these differences were statistically significant (*p* < 0.05).

**Figure 1:**
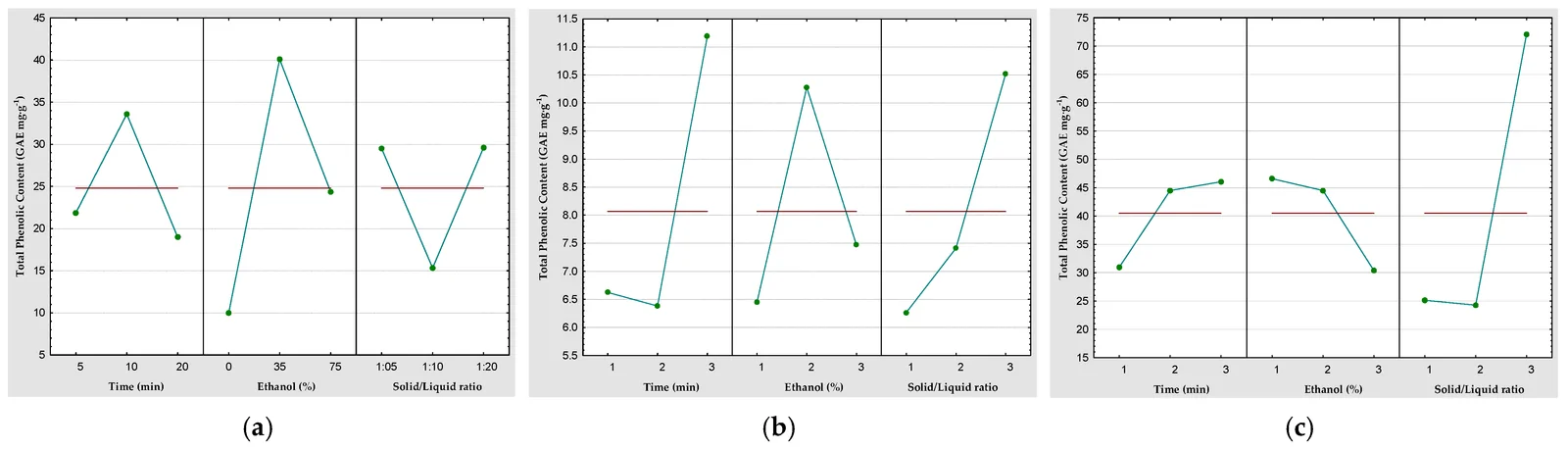
Individual factors performance at different levels: (**a**) *Flourensia cernua*; (**b**) *Artemisia ludoviciana*; (**c**) *Phoradendron californicum*. The red lines correspond to the means, and the green dots represent the behavior of the levels within each factor.

Figure 1 illustrates the effect of the evaluated factors on TPC in the extracts of the three species studied. For *F. cernua* (Figure 1a), levels 1 and 3 of extraction time and ethanol concentration resulted in lower TPC, whereas level 2 produced the highest response for both factors. The solid/liquid ratio showed similar responses at levels 1 and 3; therefore, level 1 was selected to reduce solvent consumption while maintaining extraction efficiency. In *A. ludoviciana* (Figure 1b), increasing the solid/liquid ratio resulted in higher TPC, indicating greater extraction efficiency with increasing solvent volume relative to the plant material. Extraction time showed the highest response at level 3, whereas ethanol concentration showed the highest response at level 2. For *P. californicum* (Figure 1c), TPC increased with extraction time and solid/liquid ratio, with level 3 showing the highest response for both factors. In contrast, level 1 of ethanol concentration resulted in the highest TPC.

Table 5 presents the percentage contribution of each factor, calculated by analysis of variance (ANOVA) for the three species. The factor most influencing TPC extraction differed among species. Ethanol concentration was the most influential factor for *F. cernua* (57.06%), while extraction time was predominant for *A. ludoviciana* (42.85%), and solid/liquid ratio showed the highest contribution for *P. californicum* (82.54%). Thus, the most contributing factor was species-specific.

### 3.2. Free Radical-Scavenging Activity

The antioxidant activity results (Table 4) showed significant differences among the three evaluated species (Tukey’s test, *p* < 0.05). In the ABTS assay, *A. ludoviciana* exhibited the highest antioxidant activity, with a half-maximal inhibitory concentration (IC_50_) of 19.37 ± 4.23 μg∙mL^−1^, followed by *F. cernua* (1985.59 ± 104.17 μg∙mL^−1^) and *P. californicum* (4016.50 ± 267.01 μg∙mL^−1^). On the other hand, no significant differences were observed in the DPPH assay between *F. cernua* (755.92 ± 70.60 μg∙mL^−1^) and *A. ludoviciana* (938.73 ± 27.29 μg∙mL^−1^). These values were significantly lower than those obtained for *P. californicum*, which had an IC_50_ value of 2526.65 ± 201.79 μg·mL^−1^.

### 3.3. Antiparasitic Activity

Table 4 presents the IC_50_ values for the parasiticidal activity of the evaluated extracts. Significant differences were observed among the three species (Tukey’s test, *p* < 0.05). *A. ludoviciana* exhibited the lowest IC_50_ value (0.51 ± 0.20 mg·mL^−1^), followed by *F. cernua* (1.16 ± 0.23 mg·mL^−1^) and *P. californicum* (2.86 ± 0.89 mg·mL^−1^). Tukey’s test indicated that *A. ludoviciana* and *F. cernua* did not differ significantly from each other (*p* > 0.05), whereas both species showed significantly lower IC_50_ values than *P. californicum*.

### 3.4. Growth-Inhibitory Activity

**Figure 2:**
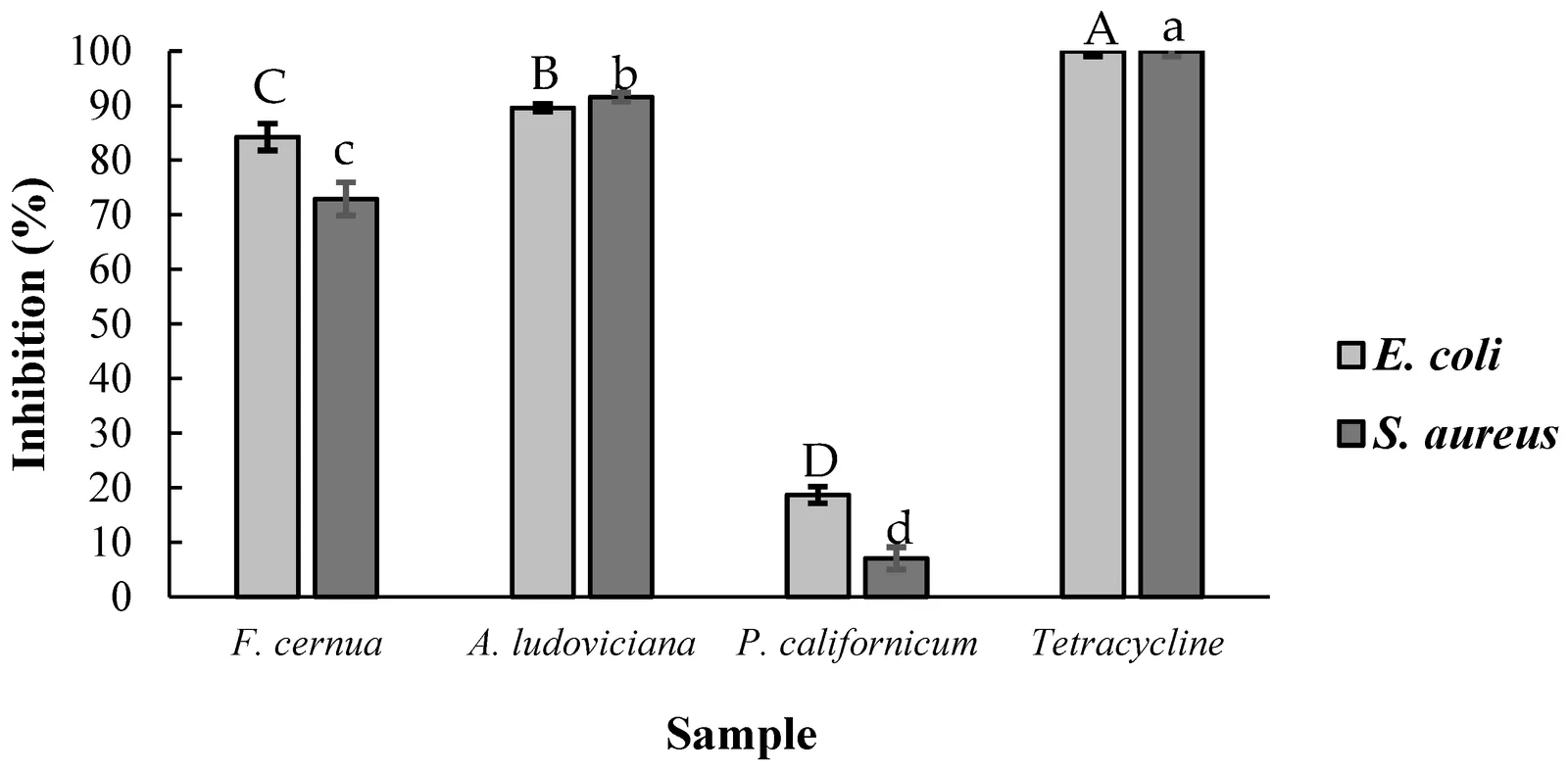
Growth-inhibitory activity of the plant extracts. Significant differences among treatments are denoted by distinct letters (Tukey’s test, *p* < 0.05): uppercase for *E. coli* and lowercase for *S. aureus*. The bacteria were analyzed separately. *F. cernua*: *Flourensia cernua*; *A. ludoviciana*: *Artemisia ludoviciana*; *P. californicum*: *Phoradendron californicum*.

Figure 2 shows the inhibitory effects of the plant species against pathogenic bacteria. Tukey’s comparison test revealed significant differences among the three plant species and the control (*p* < 0.05). Tetracycline (128 µg/mL), used as the positive control, achieved 100% inhibition against both bacterial strains. Among the plant extracts, *A. ludoviciana* exhibited the highest inhibitory activity, reaching 89.59 ± 0.71% against *E. coli* and 91.55 ± 0.84% against *S. aureus*. This was followed by *F. cernua*, which showed inhibition rates of 84.25 ± 2.45% and 72.90 ± 3.01% against *E. coli* and *S. aureus*, respectively. In contrast, *P. californicum* exhibited the lowest inhibitory activity, with inhibition rates of 18.63 ± 1.52% against *E. coli* and 7.01 ± 2.05% against *S. aureus*. None of the tested extracts reached the MIC within the concentration range evaluated.

### 3.5. HPLC-MS Characterization

Table 6 presents the compounds tentatively identified in each extract, along with their retention times and mass spectral data. In total, 12 compounds were tentatively identified in *F. cernua*, 10 in *A. ludoviciana*, and 8 in *P. californicum*. Hydroxycinnamic acid derivatives were the main compound class tentatively identified across the three species.

## 4. Discussion

During the optimization of the extraction process for *F. cernua* (Figure 1a), ethanol concentration at level 2 was the factor with the greatest influence on TPC. This behavior may be attributed to the ability of the solvent system to promote the recovery of specific compounds according to their polarity and solubility. In this regard, solvent composition plays a critical role in determining the affinity between the extraction medium and the compounds present in the plant matrix [20]. For *A. ludoviciana*, extraction time at level 3 had the greatest influence on the response (Figure 1b). This effect may be associated with the acoustic cavitation generated during ultrasound application, which can promote an increase in the temperature of the extraction medium. Depending on the thermal stability of the target compounds, increased temperature and prolonged exposure may enhance the release of phenolic compounds or, conversely, promote their degradation [21]. In contrast, for *P. californicum*, the solid-to-liquid ratio at level 3 was the factor with the greatest influence on the extraction response (Figure 1c). This behavior suggests that a higher solvent-to-solid proportion may favor the mass transfer and solubilization of phenolic compounds, potentially because the larger volume of extraction medium provides greater capacity to maintain the compounds released from the plant matrix in solution [22].

The TPC obtained under the optimal extraction conditions for *F. cernua* was lower than that reported by Aranda-Ledezma et al. [15], who obtained 132.75 ± 1.91 GAE mg·g^−1^ using UAE. This difference may be related to differences in the extraction procedures employed in the two studies, particularly the purification step performed by these authors, which may have removed potentially interfering compounds and concentrated the target fractions, thereby resulting in higher TPC. Similarly, the total flavonoid and condensed tannin contents obtained in the present study were lower than those reported by Aguirre-García et al. [23], who reported values of 124.80 ± 6.90 CE mg·g^−1^ and 100.20 ± 13.20 CE mg·g^−1^, respectively. These differences may be attributed to the combination of UAE and MAE employed by these authors, as the sequential or combined application of these technologies may enhance plant matrix disruption and consequently promote the release of phenolic compounds [24]. The *A. ludoviciana* extracts exhibited similar TPC and total flavonoid contents compared with those reported by Fernández-Cortéz et al. [25], who obtained values of 14.40 ± 0.00 GAE mg·g^−1^ and 2.90 ± 0.90 QE mg·g^−1^, respectively. This difference may be attributed to the plant material used by these authors, which was obtained under ex vitro conditions following a micropropagation process. The hardening and acclimatization stages associated with micropropagation may induce physiological responses that alter the biosynthesis and accumulation of phytochemicals. Assanga et al. [26] reported TPC, condensed tannin, and total flavonoid contents of 227.00 GAE mg·g^−1^, 150.00 CE mg·g^−1^, and 130.00 CE mg·g^−1^, respectively, in *P. californicum*, using maceration for 3 days with absolute ethanol and the whole plant as the raw material. The differences observed compared with the present study may be attributed to the longer solvent–plant contact time and the use of the entire plant, including stems, which may increase phenolic compound recovery.

The antioxidant activity was higher in the *A. ludoviciana* extracts according to the ABTS assay, exceeding the value reported by Alfaro-Jiménez et al. [27], who obtained an IC_50_ of 187.13 μg·mL^−1^ using a 15 min infusion at boiling temperature. This difference may be associated with the extraction conditions, as prolonged exposure to elevated temperatures may affect the stability of certain antioxidant compounds. In contrast, *F. cernua* exhibited the highest activity in the DPPH assay; however, this activity was lower than that reported by Rodríguez et al. [28], who obtained IC_50_ values of 174.37 ± 6.58 μg·mL^−1^ for DPPH and 69.27 ± 8.95 μg·mL^−1^ for ABTS using Soxhlet extraction for 72 h. Although these values were higher, the extraction process required considerably more time, whereas UAE enabled antioxidant activity to be obtained within shorter extraction periods, highlighting its potential as an alternative approach to improve extraction process efficiency.

The antiparasitic activity of *A. ludoviciana* has been demonstrated and has been associated with its essential oils. Ezeta-Miranda et al. [29] demonstrated that ethyl acetate extracts exhibited ovicidal activity against *Fasciola hepatica*, as well as activity against adult parasites. In addition, the antileishmanial potential of *A. ludoviciana* has been reported, as its essential oils obtained through hydrodistillation achieved 100% inhibition of *Leishmania infantum* promastigotes within 14 h [30]. Although previous studies have primarily focused on essential oils, our research group has also determined the antileishmanial activity of the plants evaluated in the present study using infusion extracts, obtaining comparable results. No significant differences were observed for *A. ludoviciana*, which exhibited an IC_50_ of 0.51 ± 0.01 mg·mL^−1^. In contrast, *P. californicum* showed lower activity (IC_50_ = 2.63 ± 0.20 mg·mL^−1^), whereas *F. cernua* exhibited higher activity, with an IC_50_ of 1.25 ± 0.20 mg·mL^−1^. This activity may be associated with phenolic compounds with potential antiparasitic activity, such as caffeoylquinic acid [31].

Scientific evidence indicates that extracts from the genus *Artemisia* exhibit antibacterial activity against a variety of pathogenic microorganisms, including *Mycobacterium tuberculosis*, *Staphylococcus aureus*, *Escherichia coli*, *Listeria monocytogenes*, and *Salmonella enteritidis*, demonstrating their potential as sources of antimicrobial agents [32,33]. Likewise, *F. cernua* has shown activity against *E. coli*, *S. aureus*, and *Candida albicans* when hydroalcoholic extracts are used [34]. In contrast, evidence regarding the antibacterial activity of *P. californicum* remains limited, although inhibitory effects against *E. coli* and *S. aureus* have been reported. The antimicrobial activity of these extracts is primarily associated with the presence of phenolic compounds, whose hydroxyl groups may interact with bacterial cellular structures and components, thereby disrupting essential processes required for bacterial growth and promoting the generation of reactive oxygen species [31]. A limitation of the antibacterial assay was that extract-only blanks were not included; therefore, the potential contribution of the intrinsic absorbance or turbidity of the extracts at 620 nm could not be independently corrected.

HPLC–MS analysis tentatively assigned rosmanol in the *A. ludoviciana* extract, a diterpene to which several biological activities have been attributed, particularly antioxidant, anti-inflammatory, anticancer, and neuroprotective effects [35]. In addition, methoxylated flavones such as hispidulin, cirsiliol, and jaceosidin were tentatively assigned. These compounds have been reported to exhibit anticancer, antioxidant, antiosteoporotic, and antiepileptic properties, as well as the ability to inhibit platelet aggregation [36,37]. In *F. cernua*, caffeic acid, gallic acid, caffeoylquinic acids, galloylquinic acids, and dicaffeoylquinic acids were tentatively identified. These compounds have been associated with a wide range of biological properties, including antioxidant, antimicrobial, anti-inflammatory, antidiabetic, antiangiogenic, antihyperglycemic, cardiovascular, hepatoprotective, and neuroprotective activities [38]. Furthermore, apigenin has demonstrated a broad spectrum of pharmacological activities, particularly anti-inflammatory, anticancer, antioxidant, radioprotective, cardiovascular, nephroprotective, and neuroprotective effects, in addition to its potential to modulate glucose and lipid metabolism [39,40]. In the *P. californicum* extract, feruloylquinic acid was tentatively identified, a compound associated with antihyperglycemic, and antioxidant effects. Its hepatoprotective activity has been associated with preventing oxidative stress-induced liver damage, whereas its antihyperglycemic effect may be related to modulating enzymes involved in carbohydrate metabolism [41].

Several compounds with diverse reported biological activities were tentatively identified; however, because the analysis was qualitative, their concentrations could not be determined, nor could a direct relationship be established between their abundance and the biological activities observed. Therefore, the presence of a greater number or diversity of bioactive compounds in an extract does not necessarily imply greater biological activity. Moreover, the observed effects may be influenced by interactions among the different metabolites present in the extracts, which may result in additive or synergistic effects that contribute to the biological activities evaluated.

## 5. Conclusions

The Taguchi L9 experimental design enabled determination of the optimal extraction conditions for the evaluated factors in UAE of hydrolyzable tannins. *F. cernua* exhibited the highest condensed tannin content, whereas *P. californicum* showed the highest total flavonoid content. The extracts also exhibited radical-scavenging activity, with *A. ludoviciana* showing the highest activity in the ABTS assay and *F. cernua* in the DPPH assay. Furthermore, the extracts exhibited inhibitory activity against the pathogenic bacteria *Staphylococcus aureus* and *Escherichia coli*, as well as against the parasite *Leishmania braziliensis*, with *A. ludoviciana* showing the strongest overall antiparasitic and antibacterial activity. HPLC–MS characterization allowed the tentative assignment of compounds potentially associated with the observed biological activities, including caffeoylquinic acid derivatives and apigenin derivatives. Overall, the results support the potential use of these extracts as sources of natural bioactive ingredients for applications in the food, cosmetic, and pharmaceutical industries. However, further studies addressing compound purification, bioavailability, toxicity, stability, and validation in appropriate biological models are required to confirm their suitability for specific industrial applications.

## Figures and Tables

**Table 1:** Factors and levels used in the Taguchi L9 orthogonal array.

Factor	Level 1	Level 2	Level 3
Time (min)	5	10	20
Ethanol (%)	0	35	70
Solid/Liquid ratio	1:5	1:10	1:20

**Table 2:** Taguchi L9 orthogonal array and corresponding concentrations of total phenolic content for *F. cernua*, *A. ludoviciana*, and *P. californicum*.

Treatment	Time	Ethanol	Solid/Liquid Ratio	GAE mg∙g^−1^		
				*F. cernua*	*A. ludoviciana*	*P. californicum*
1	1	1	1	6.15 ± 0.08	2.37 ± 0.42	21.02 ± 1.26
2	1	2	2	34.37 ± 2.08	9.10 ± 0.65	21.03 ± 0.58
3	1	3	3	25.07 ± 0.50	8.41 ± 0.31	50.73 ± 5.03
4	2	1	2	8.17 ± 0.44	4.04 ± 0.06	32.70 ± 2.00
5	2	2	3	48.07 ± 5.03	10.21 ± 0.46	79.40 ± 3.46
6	2	3	1	44.52 ± 2.75	4.90 ± 0.03	21.35 ± 0.87
7	3	1	3	15.67 ± 0.61	12.94 ± 0.69	86.07 ± 3.06
8	3	2	1	37.85 ± 1.50	11.52 ± 0.76	33.02 ± 2.08
9	3	3	2	3.44 ± 0.35	9.10 ± 0.15	19.03 ± 2.52

*F. cernua*: *Flourensia cernua*; *A. ludoviciana*: *Artemisia ludoviciana*; *P. californicum*: *Phoradendron californicum*.

**Table 3:** Predicted and experimentally observed responses under the optimal extraction conditions obtained using the Taguchi model.

Sample	Time (min)	Ethanol (%)	Solid/Liquid Ratio	GAE mg∙g^−1^	
				Predicted Values	Observed Values
*F. cernua*	10	35	1:5	53.66	57.40 ± 4.00
*A. ludoviciana*	20	35	1:20	15.85	17.81 ± 1.70
*P. californicum*	20	0	1:20	87.33	106.07 ± 4.16

*F. cernua*: *Flourensia cernua*; *A. ludoviciana*: *Artemisia ludoviciana*; *P. californicum*: *Phoradendron californicum*.

**Table 4:** Antioxidant and antiparasitic activities, total flavonoids, and condensed tannins of extracts from three plant species.

Sample	ABTS (IC_50_ μg∙mL^−1^)	DPPH (IC_50_ μg∙mL^−1^)	Antiparasitic Activity (IC_50_ mg∙mL^−1^)	Total Flavonoids (CE mg∙g^−1^)	Condensed Tannins (CE mg∙g^−1^)
*F. cernua*	1985.59 ± 104.17 ^b^	755.92 ± 70.60 ^a^	1.16 ± 0.23 ^ab^	7.28 ± 0.39 ^b^	6.71 ± 0.10 ^a^
*A. ludoviciana*	19.37 ± 4.23 ^a^	938.73 ± 27.29 ^a^	0.51 ± 0.20 ^a^	5.06 ± 0.04 ^c^	4.46 ± 0.11 ^c^
*P. californicum*	4016.50 ± 267.01 ^c^	2526.65 ± 201.79 ^b^	2.86 ± 0.89 ^b^	9.04 ± 0.31 ^a^	5.29 ± 0.07 ^b^

*F. cernua*: *Flourensia cernua*; *A. ludoviciana*: *Artemisia ludoviciana*; *P. californicum*: *Phoradendron californicum*; CE: catechin equivalents. Different superscript letters within the same column indicate significant differences between plant species (Tukey’s test, *p* < 0.05).

**Table 5:** Analysis of variance (ANOVA) by species.

Sample	Factors	SS	df	MS	F	*p*	Contribution (%)
*Flourensia* *cernua*	Time	1076.24	2	538.12	13.82	0.00	15.05
	Ethanol	4078.97	2	2039.48	52.39	0.00	57.06
	Solid/Liquid ratio	1215.14	2	607.57	15.61	0.00	17.00
	Error	778.51	20	38.93			10.89
	Total	7148.85	26				100.00
*Artemisia* *ludoviciana*	Time	131.84	2	65.92	73.49	0.00	42.85
	Ethanol	70.68	2	35.34	39.40	0.00	22.97
	Solid/Liquid ratio	87.20	2	43.60	48.61	0.00	28.34
	Error	17.94	20	0.90			5.83
	Total	307.66	26				100.00
*Phoradendron californicum*	Time	1243.56	2	621.78	60.37	0.00	7.62
	Ethanol	1400.22	2	700.11	67.97	0.00	8.58
	Solid/Liquid ratio	13,469.77	2	6734.88	653.87	0.00	82.54
	Error	206.00	20	10.30			1.26
	Total	16,319.55	26				100.00

SS: sum of squares; df: degrees of freedom; MS: mean square.

**Table 6:** Tentative identification of compounds in the extracts by HPLC-MS.

Sample	Retention Time (min)	Mass [M−H]^−^	Compound	Family
*Flourensia cernua*	6.533	340.9	Caffeic acid 4-O-glucoside	Hydroxycinnamic acids
	7.529	352.9	1-Caffeoylquinic acid	Hydroxycinnamic acids
	24.551	332.9	Gallic acid 4-O-glucoside	Hydroxybenzoic acids
	26.732	592.8	Apigenin 6,8-di-C-glucoside	Flavones
	28.985	562.8	Apigenin arabinoside-glucoside	Flavones
	37.601	342.8	5-O-Galloylquinic acid	Hydroxybenzoic acids
	40.528	514.8	1,3-Dicaffeoylquinic acid	Hydroxycinnamic acids
	43.434	312.9	Cirsimaritin	Methoxyflavonols
	47.182	344.9	Rosmanol	Hydroxycinnamic acids
	48.693	268.8	Apigenin	Flavones
	51.221	243	Piceatannol	Stilbenes
	52.698	328.9	3,7-Dimethylquercetin	Methoxyflavonols
*Artemisia ludoviciana*	7.49	190.8	Scopoletin	Hydroxycoumarins
	27.902	352.8	1-Caffeoylquinic acid	Hydroxycinnamic acids
	31.301	386.8	Medioresinol	Lignans
	37.444	352.7	3-Caffeoylquinic acid	Hydroxycinnamic acids
	39.193	514.7	1,3-Dicaffeoylquinic acid	Hydroxycinnamic acids
	44.869	314.8	Rhamnetin	Methoxyflavonols
	46.849	344.8	Rosmanol	Phenolic terpenes
	49.029	298.8	Hispidulin	Methoxyflavones
	51.126	328.8	Jaceosidin	Methoxyflavones
	54.653	342.8	Cirsilineol	Methoxyflavones
*Phoradendron californicum*	7.213	190.9	Scopoletin	Hydroxycoumarins
	10.064	384.8	5-5′-Dehydrodiferulic acid	Methoxycinnamic acid dimers
	15.843	352.9	1-Caffeoylquinic acid	Hydroxycinnamic acids
	24.26	336.9	3-p-Coumaroylquinic acid	Hydroxycinnamic acids
	25.216	366.9	3-Feruloylquinic acid	Methoxycinnamic acids
	28.572	592.9	Apigenin 6,8-di-C-glucoside	Flavones
	31.573	562.9	Apigenin arabinoside-glucoside	Flavones
	34.855	430.9	Apigenin 6-C-glucoside	Flavones

## Data Availability

The raw data supporting this study are included in the article. Further inquiries can be directed to the corresponding authors.

## Abbreviations

The following abbreviations are used in this manuscript:

UAE	Ultrasound-Assisted Extraction
TPC	Total Phenolic Content
IC_50_	Half-Maximal Inhibitory Concentration
HPLC-MS	High-Performance Liquid Chromatography and Mass Spectroscopy
